# Incidence of Medically-Attended Norovirus-Associated Acute Gastroenteritis in Four Veteran’s Affairs Medical Center Populations in the United States, 2011-2012

**DOI:** 10.1371/journal.pone.0126733

**Published:** 2015-05-21

**Authors:** Scott P. Grytdal, David Rimland, S. Hannah Shirley, Maria C. Rodriguez-Barradas, Matthew Bidwell Goetz, Sheldon T. Brown, Cynthia Lucero-Obusan, Mark Holodniy, Christopher Graber, Umesh Parashar, Jan Vinjé, Ben Lopman

**Affiliations:** 1 Division of Viral Diseases, Centers for Disease Control and Prevention, Atlanta, Georgia, United States of America; 2 Atlanta VA Medical Center and Emory University School of Medicine, Atlanta, Georgia, United States of America; 3 Atlanta Research and Education Foundation, Decatur, Georgia, United States of America; 4 Infectious Diseases Section, Michael E. DeBakey VA Medical Center and Department of Medicine, Baylor College of Medicine, Houston, Texas, United States of America; 5 VA Greater Los Angeles Healthcare System and David Geffen School of Medicine at UCLA, Los Angeles, California, United States of America; 6 James J. Peters VA Medical Center, Bronx, New York, United States of America; 7 VA Office of Public Health Washington, D.C./Palo Alto, California, United States of America; 8 Stanford University School of Medicine, Stanford, California, United States of America; Beijing Institute of Microbiology and Epidemiology, CHINA

## Abstract

An estimated 179 million acute gastroenteritis (AGE) illnesses occur annually in the United States. The role of noroviruses in hospital-related AGE has not been well-documented in the U. S. We estimated the population incidence of community- acquired outpatient and inpatient norovirus AGE encounters, as well as hospital-acquired inpatient norovirus AGE among inpatients at four Veterans Affairs (VA) Medical Centers (VAMCs). Fifty (4%) of 1,160 stool specimens collected ≤7 days from symptom onset tested positive for norovirus. During a one year period, the estimated incidence of outpatient, community- and hospital-acquired inpatient norovirus AGE was 188 cases, 11 cases, and 54 cases/ 100,000 patients, respectively. This study demonstrates the incidence of outpatient and community- and hospital-acquired inpatient norovirus AGE among the VA population seeking care at these four VAMCs.

## Introduction

An estimated 179 million acute gastroenteritis (AGE) illnesses occur each year in the United States [1]. While pediatric hospital admissions for AGE have markedly decreased in recent years due to the introduction of rotavirus vaccines in the United States, AGE hospitalization has been increasing in incidence among adults [2]. Noroviruses are also increasingly recognized as a frequent cause of sporadic AGE disease and outbreaks, estimated to cause 21 million cases of AGE, 2.1 million ambulatory visits, and 71,000 hospitalizations annually in the U.S. [1–4]. Although norovirus AGE is generally self-limiting in otherwise healthy adults, vulnerable populations can be more severely affected by norovirus AGE, and norovirus may contribute to mortality among elderly persons [5–7].

Sixty-four percent of AGE outbreaks attributed to noroviruses reported to a passive U.S. surveillance system from 2009–2010 occurred in healthcare settings, primarily nursing homes [8]. The admission of patients for norovirus and infection among visitors and staff both pose a risk for transmission of norovirus gastroenteritis in the healthcare setting. The incidence of noroviruses in healthcare-related AGE has not been well-documented in U.S. acute care facilities and much needs to be known about norovirus disease in this setting in order to appropriately prioritize prevention and control activities. We aimed to estimate the population-based incidence of community-acquired outpatient and inpatient encounters for norovirus AGE, as well as hospital-acquired norovirus AGE incidence among inpatients in four VAMCs.

## Methods

This study was conducted at four large and geographically dispersed VAMCs in four states (Atlanta VA Medical Center, Atlanta, GA; Michael E. DeBakey VA Medical Center, Houston, TX; Greater Los Angeles VA Healthcare System, Los Angeles, CA; and James J. Peters VA Medical Center, Bronx, NY). Each site was selected based on their willingness to participate in the study; their representativeness to other VAMCs was not assessed prior to initiating the study. Patient names were removed from data and specimens prior to being sent to the Centers for Disease Control and Prevention (CDC). This study protocol was reviewed and approved by the Institutional Review Boards (IRB) at CDC and at each of four VAMCs.

From November 1, 2011 to October 31, 2012, all stool specimens from clinician-requested diagnostic testing in which there was remaining material were stored and shipped to CDC for supplemental testing for the presence of norovirus. Whole stool specimens were immediately stored at -70°C or were kept refrigerated at 4°C until they were shipped on dry ice to CDC. Specimens were then stored at 4°C at CDC until testing was performed. All specimens were tested for the presence of norovirus RNA by using the AgPath-ID One-Step RT-PCR Kit (Applied Biosystems, Foster City, CA, USA) on the 7500 Realtime PCR platform (Applied Biosystems) [9]. Norovirus-positive specimens were genotyped by comparing region C sequences to reference strains using phylogenetic analysis as per CaliciNet protocols [9]. Specimens collected from the same patient within 7 days of another collected specimen were excluded from analyses.

Clinical and epidemiological data associated with each stool specimen were extracted from the VAMC electronic medical record system using a standardized form. These data included age and sex of the patient; presence of vomiting or diarrhea; dates of specimen collection, symptom onset, and admission (for inpatient specimens); if gastroenteritis was a specific reason for admission, and testing results for other pathogens from the VAMC hospital laboratory.

Specimens collected ≤7 days from symptom onset were included in the epidemiologic analysis; those collected >7 days after symptom onset were excluded since viruses are less likely to be the cause of illness or detected after 7 days [10]. Norovirus AGE is unlikely to last for more than one week and patients with a sample taken more than 7 days after onset are unlikely to be a result of norovirus. Second, while norovirus shedding can persist, viral loads decrease over the course of illness so detection rates decrease substantially after 7 days. Cases were categorized based on the following definitions: 1) *outpatient*, specimen collected from an individual not known to be admitted to a VAMC; 2) *community-acquired inpatient*, specimen from individual admitted to a VAMC with symptom onset on or before the admission date; 3) *hospital-acquired inpatient*, specimen from an individual admitted to a VAMC with symptom onset 1 or more days after date of admission.

Each VAMC provided the total unique patients served at their facility during January-December 2012 and these numbers were used as the denominator for outpatient and community-acquired inpatient incidence calculations. The total ‘unique patients served’ includes all veterans who utilize any VA services and is therefore the best indicator of population denominator. In addition, each VAMC provided the total inpatient discharges and outpatient encounters during November 2011 to October 2012. AGE discharge diagnoses for inpatients and outpatient encounters were extracted and defined using a previously described set of ICD9-CM codes [2]. We considered an outpatient encounter or inpatient discharge to be an episode of AGE if any of these ICD9-CM codes were present at either the admission code or in any discharge code position.

### Incidence calculations

Norovirus incidence among VAMC patients seeking care was calculated on the basis of norovirus prevalence in outpatient or inpatient specimens (*p(noro*_*i*_ *)*), number of AGE outpatient encounters or inpatient discharges (*E*_*i*_), and the number of unique patients served (N) or total number of hospital discharges (*A*). Outpatient norovirus incidence per 100,000 population was calculated as

$$\left(10^{5}*p{\left(noro\right)}_{out}*E_{out}\right)/\;N$$

Community-acquired inpatient norovirus incidence per 100,000 patients was calculated as

$$\left(10^{5}*p{\left(noro\right)}_{in\text{-}com}*p(com)*E_{in}\right)/\;N$$

where p(noro)_in-com_ is the prevalence of norovirus amongst community-acquired inpatient AGE specimens, and *p(com)*, the proportion of total inpatient discharges that were community-acquired, was calculated as:

p(com)=C/(H+C),

where *C* represents the number of inpatient specimens that were classified as community-acquired infections and *H* represents the number of inpatient specimens that were classified as hospital-acquired infections.

For hospital-acquired norovirus incidence was calculated as

$$\left(10^{5}*p{\left(noro\right)}_{in\text{-}hosp}*p(hosp)*E_{in}\right)/\;A,$$

where p(noro)_in-hosp_ is the prevalence of norovirus amongst hospital-acquired inpatient AGE specimens; and

p(hosp)=1-p(com);

and *A* = the number of hospital inpatient discharges (as opposed to a population rate)

Incidence calculations were also performed stratified by age (at 65 years) and by VAMC site.

## Results

A total of 2877 specimens were collected from the four VAMCs, of which 86 (3%) were collected from the same patient within 7 days of another collected specimen and were excluded from analyses. Fifty-eight percent (n = 1,631) were collected 8 or more days after the onset of symptoms and were also excluded from subsequent analysis, leaving a final total of 1,160 specimens that were included in analyses. Twenty-five percent of specimens originated from Site A, 20% from Site B, 45% from Site C, and 11% from Site D. Diarrhea and vomiting were reported to be present in 87% and 20%, respectively, of patients whose stools were included in analyses. Eighty-two percent of specimens were collected from inpatients, and 28% of all inpatient specimens were from patients who were admitted to VAMCs specifically for gastroenteritis as reported by the VAMC. Thirty-nine percent (n = 375) of inpatient specimens were categorized as inpatient community-acquired and 61% (n = 582) were inpatient hospital-acquired. The median age of patients from whom specimens were collected was 64 years (range: 20–95 years). VAMCs tested 1,032 (89%) of specimens for the presence of *Clostridium difficile* toxin, 481 (41%) were tested for the presence of other enteric bacteria, and 325 (28%) were tested for the presence of ova and parasites.

**Fig 1:**
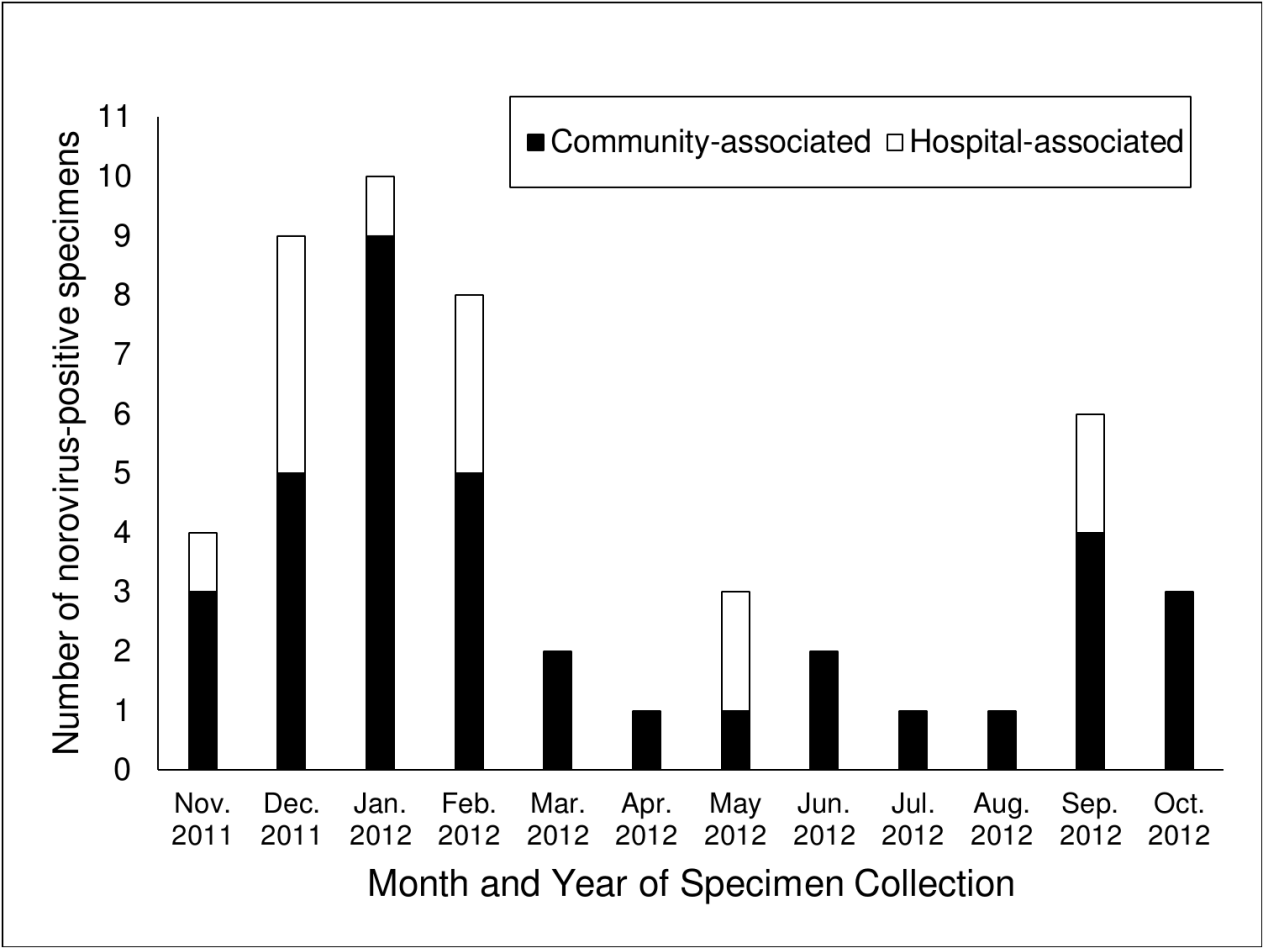
Number of norovirus-positive specimens, by source of infection; 4 VAMCs, November 2011-October 2012.

Fifty (4%) specimens of 1,160 specimens tested positive for norovirus. Seven percent (14/203) of outpatient specimens were norovirus-positive, as well as 6% (23/375) of community-acquired inpatient specimens and 2% (13/582) of hospital-acquired inpatient (n = 582) specimens. Eighteen percent (n = 9) of norovirus-positive specimens also tested positive for another pathogen that causes AGE, including 5 (10%) that tested positive for *C*. *difficile* toxin. Norovirus-positivity followed a seasonal pattern with 33 (66%) of norovirus-positive specimens collected between November 2011 and March 2012 (Fig 1).

The overall estimated population incidence of community-acquired norovirus-associated AGE requiring medical care was 200 cases/100,000 patients, with outpatient and inpatient incidence of 188 and 11 cases/100,000 patients, respectively (S1 and S2 Tables). Incidence was similar between patients under 65 years and those over 65 years of age (outpatient: 172 cases per 100,000 patients vs. 200 cases per 100,000 patients (S1 Table); community-acquired inpatient: 8 cases per 100,000 patients vs. 14 cases per 100,000 patients (S2 Table); hospital-acquired inpatient: 45 cases per 100,000 patients vs. 66 cases per 100,000 patients (S3 Table)). The population incidence of outpatient norovirus-associated AGE was notably lower at Site B compared to other sites.

The overall estimated incidence of hospital-acquired norovirus-associated AGE was 54 cases/100,000 inpatient discharges (S3 Table). The incidence of hospital-acquired norovirus-associated AGE varied by site and was much higher among patients at Site D (261 cases per 100,000 discharges compared to 54 cases per 100,000 discharges overall). This site reported the single outbreak that was detected during this study. From November 29, 2011 to January 12, 2012, 40 patients were identified with nausea, vomiting, or diarrhea during their hospital stay and were consequently placed in single-occupancy rooms. Thirty-three (83%) cases displayed AGE symptoms 48 hours or more after admission, indicating their infection was indeed acquired at the hospital. The remaining seven (18%) cases displayed symptoms less than 48 hours after admission and were considered to have acquired a norovirus infection prior to hospital admission. Stool specimens were collected for 7 (18%) of the 40 cases; 4 (60%) of these specimens tested positive for norovirus.

**Fig 2:**
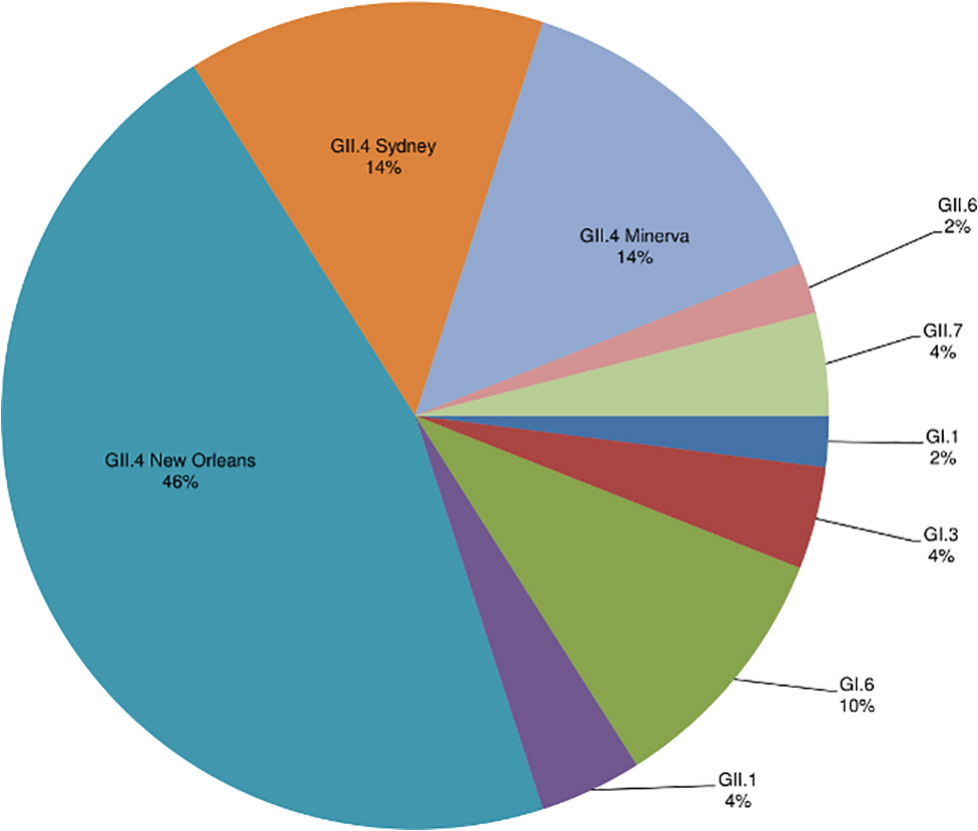
Distribution of norovirus genotypes among specimens from 4 VAMCs; November 2011-October 2012 (n = 50).

A total of 7 norovirus genotypes were detected among all norovirus-positive specimens, of which the majority (74%) were typed as one of the GII.4 variants including GII.4 New Orleans (46%), GII.4 Minerva (now known as GII.4 Den Haag) (14%), and GII.4 Sydney (14%) (Fig 2). Five specimens (10%) were typed as GI.6, an uncommon genotype that emerged nationwide in this period, then subsequently became less common [11].

## Discussion

This study provides estimates of laboratory-confirmed population-based community-acquired norovirus AGE requiring outpatient and inpatient care as well as hospital-acquired inpatient incidence by using fecal specimens collected during routine patient care in four VAMCs. Noroviruses were detected in 7% of outpatient and 6% of community-acquired inpatient AGE specimens tested, resulting in an estimated incidence of 188 cases per 100,000 patients and 12 cases per 100,000 discharges, respectively. Norovirus-positive specimens followed a winter seasonal distribution, and norovirus strains identified in this study were consistent with strains found in earlier studies conducted in this period and with novel strains associated with outbreaks reported during 2011–2012 [9, 11, 12].

The overall proportion of norovirus-positive specimens identified in our study (2 to 7%) is similar to findings by Hall et al., in which specimens submitted by patients who sought care at a health maintenance organization in Georgia, USA were tested for norovirus[10]. The low detection rates in both of these studies are likely a result of a passive and convenient design whereby fecal specimens from patients with AGE submitted for routine stool testing were examined for the presence of norovirus. Our norovirus-associated AGE outpatient incidence (188 cases/100,000 population) was markedly lower than other estimates derived from patients seeking care for AGE in the United States (570–640 cases/100,000 population) [4, 10], the UK (210–540 cases/100,000 population) [13, 14], the Netherlands (920 cases/100,000 population) [15], and Germany (620 cases/100,000 population) [16]. However, all of these estimates were for the whole age range, including young children where incidence is highest. Our estimate is also lower than incidence estimates among adults in the UK (260 to 380 per 100,000 population) [13, 14] and other studies in the U.S. (347 to 540 per 100,000 population) [3, 10].

Previous studies examining U.S. norovirus-associated AGE hospitalization rates have attributed the proportion of AGE hospitalization rates caused by norovirus infection. Our results show that utilizing laboratory-confirmed surveillance to estimate AGE hospitalization rates yields similar results when compared to these earlier statistical model-based estimates. For example, our rate of community-acquired norovirus-requiring inpatient care (12 cases/100,000 patients) is broadly similar to the rates estimated by indirect approaches for the United States (19 to 24/100,000 population) [2, 3] and the Netherlands (12 per 100,000 population) [15].

Utilizing laboratory-confirmed surveillance also allowed us to calculate a hospital-acquired norovirus-associated AGE incidence in a U.S. setting (at 54 norovirus cases per 100,000 hospital discharges). While the number of norovirus-positive specimens associated with hospital-acquired infections was low, the incidence was highly variable, with no cases detected in one site to 261 cases per 100,000 hospital discharges at Site D where a nosocomial outbreak of norovirus occurred in late 2011. Hospital-acquired norovirus AGE incidence is likely highly sensitive to the presence of norovirus outbreaks in healthcare facilities, and additional research is needed to more completely ascertain incidence in healthcare facilities.

The primary limitation of this study stems from the use of routinely collected specimens for bacterial or parasite diagnostic testing, rather than active surveillance for AGE. In addition, stool collection from patients suspected to have viral AGE may be less likely to be ordered due to treatment limitations. A standardized definition of AGE was not used and specimens may have been obtained from patients who had noninfectious or chronic gastroenteritis. Indeed, we suspect that a substantial number of specimens collected from patients with noninfectious or chronic gastroenteritis were included in our analyses due to the low prevalence (20%) of vomiting among patients. Additionally, the number of inpatient specimens provided by some sites sometimes exceeded the reported total number of AGE discharges. Conversely, the number of ICD-9-coded inpatient and outpatient AGE-related discharges and encounters may not reflect the true number of discharges and encounters. In addition, we used time of disease onset as an indicator of the origin of norovirus infection (community- or hospital-acquired). Extracting time of onset of specific symptoms from electronic medical records is not straightforward, especially if they were not the primary reason for admission. Therefore, it is possible that some misclassification of the origin of infection may have occurred. We found that hospital-acquired incidence estimates were somewhat sensitive to defining such cases as occurring two or more days after hospital admission (38 cases/100,000 inpatient discharges vs. 54 cases/100,000 discharges). We chose to use specimens with a reported symptom onset one or more days after admission due to the fact that the median incubation period of 1.2 days [17]. The VAMCs that conducted this study include only four of the 151 VAMCs in the United States, so there may be important differences in terms of geography, race/ethnicity and sex in our study population relative to the entire population served by VAMCs. To be sure, there are clear differences between the VA population and the broader US population in that 90% of the VA population is male, 83% is white non-Hispanic ethnicity; 43% is aged 65 years or older. In addition to these sociodemographic differences, care-seeking behavior likely differs between the VA and general US population. Additionally, the four participating VAMCs are classified as “high complexity” VAMC facilities, meaning they have high patient volumes, high levels of teaching and research, and the largest number and breadth of physician specialists. The participating VAMCs had nearly twice the average number of daily outpatient visits and discharges when compared to all VAMCs; therefore, the incidence of norovirus AGE among participating VAMCs may not be representative of all VAMCs.

This study demonstrates the incidence of community-acquired norovirus requiring outpatient or inpatient care as well as hospital-acquired norovirus AGE at 4 VAMCs during a one year period. Enhanced, active surveillance using clear case definitions and specimen sampling schemes are needed in order to better define the disease burden in the VA population.

## Supporting Information

S1 Table Estimated outpatient norovirus-associated AGE incidence at 4 VAMCs; November 2011-October 2012. (DOCX)

S2 Table Estimated community-acquired inpatient norovirus-associated AGE incidence at 4 VAMCs; November 2011-October 2012. (DOCX)

S3 Table Estimated hospital-acquired inpatient norovirus-associated AGE incidence at 4 VAMCs; November 2011-October 2012. (DOCX)

S1 Dataset Patient demographic and clinical gastroenteritis data gathered from four Veteran’s Affairs Medical Centers. (XLSX)
