# Supplementary material for: Incidence of Medically-Attended Norovirus-Associated Acute Gastroenteritis in Four Veteran’s Affairs Medical Center Populations in the United States, 2011-2012
Source: PLoS One. 2015 May 21;10(5):e0126733. doi: 10.1371/journal.pone.0126733 (PMC4440768; doi:10.1371/journal.pone.0126733)
Supplement: S1 Table — (DOCX) [file pone.0126733.s001.docx]

|  | **Symbol** | **Site** | | | | **Age category** | | **Total** |
| --- | --- | --- | --- | --- | --- | --- | --- | --- |
|  |  | **A** | **B** | **C** | **D** | **< 65 years** | **≥65 years** |  |
| Total unique patients served | *N* | 24,907 | 85,943 | 95,499 | 83,799 | 168,903 | 121,245 | 290,148 |
| AGE-related outpatient encounters | *E_out_* | 568 | 2447 | 2522 | 2361 | 5266 | 2632 | 7898 |
| Norovirus prevalence  (Positive/Total Specimens) | *p(noro)_out_* | 0.125  (5/40) | 0.022 (2/91) | 0.116  (5/43) | 0.069  (2/29) | 0.055 (7/127) | 0.092  (7/76) | 0.069  (14/203) |
| Norovirus-associated outpatient encounters per 100,000 patients |  | 285 | 63 | 307 | 194 | 172 | 200 | 188 |
