# Supplementary material for: Incidence of Medically-Attended Norovirus-Associated Acute Gastroenteritis in Four Veteran’s Affairs Medical Center Populations in the United States, 2011-2012
Source: PLoS One. 2015 May 21;10(5):e0126733. doi: 10.1371/journal.pone.0126733 (PMC4440768; doi:10.1371/journal.pone.0126733)
Supplement: S2 Table — (DOCX) [file pone.0126733.s002.docx]

|  | **Symbol** | **Site** | | | | **Age category** | | **Total** |
| --- | --- | --- | --- | --- | --- | --- | --- | --- |
|  |  | **A** | **B** | **C** | **D** | **< 65 years** | **≥65 years** |  |
| Total unique patients served | *N* | 24,907 | 85,943 | 95,499 | 83,799 | 168,903 | 121,245 | 290,148 |
| AGE-related inpatient discharges | *E_in_* | 172 | 401 | 454 | 271 | 705 | 593 | 1298 |
| Proportion of specimens from community-acquired infection | *p(com)* | 0.41 (102/247) | 0.46  (65/140) | 0.35 (166/477) | 0.45  (42/93) | 0.44  (217/488) | 0.34  (158/469) | 0.39  (375/957) |
| Norovirus prevalence  (Positive/Total Specimens) | *p(noro)_in_* | 0.030  (3/102) | 0.031  (2/65) | 0.054  (9/166) | 0.21  (9/42) | 0.046  (10/217) | 0.082  (13/158) | 0.061  (23/375) |
| Community-acquired inpatient norovirus-associated discharges per 100,000 patients |  | 9 | 7 | 9 | 31 | 8 | 14 | 11 |
