# Supplementary material for: Incidence of Medically-Attended Norovirus-Associated Acute Gastroenteritis in Four Veteran’s Affairs Medical Center Populations in the United States, 2011-2012
Source: PLoS One. 2015 May 21;10(5):e0126733. doi: 10.1371/journal.pone.0126733 (PMC4440768; doi:10.1371/journal.pone.0126733)
Supplement: S3 Table — (DOCX) [file pone.0126733.s003.docx]

|  | **Symbol** | **Site** | | | | **Age category** | | **Total** |
| --- | --- | --- | --- | --- | --- | --- | --- | --- |
|  |  | **A** | **B** | **C** | **D** | **< 65 years** | **≥65 years** |  |
| Total inpatient discharges | *A* | 3,888 | 7,211 | 13,540 | 7,830 | 19,109 | 13,360 | 32,469 |
| AGE-related inpatient discharges | *E_in_* | 172 | 401 | 454 | 271 | 705 | 593 | 1298 |
| Proportion of specimens from hospital-acquired infection | *p(hosp)* | 0.59  (145/247) | 0.54 (75/140) | 0.65 (311/477) | 0.55  (51/93) | 0.56 (271/488) | 0.66 (311/469) | 0.61 (582/957) |
| Norovirus prevalence  (Positive/Total Specimens) | *p(noro)_in-hosp_* | 0.014  (2/145) | 0  (0/75) | 0.013  (4/311) | 0.137  (7/51) | 0.022  (6/271) | 0.023 (7/311) | 0.022  (13/582) |
| Hospital-acquired norovirus-associated AGE per 100,000 inpatient discharges |  | 36 | 0 | 28 | 261 | 45 | 66 | 54 |
